# Does Your Hat Speak Your Mind? Personality Traits and Aesthetic Preferences for Hats Among Italian Young Adults

**DOI:** 10.3390/bs16020290

**Published:** 2026-02-18

**Authors:** Elena Capitani, Ivana Bianchi, Roberto Burro

**Affiliations:** 1 Department of Humanities, University of Macerata, 62100 Macerata, Italy; 2 Department of Human Sciences, University of Verona, 37129 Verona, Italy

**Keywords:** personality traits, big five, aesthetic preferences, visual preferences, hats

## Abstract

The relationship between personality and the various aspects of human cognition that influence behavior has long been recognized as central to understanding individual differences. The study investigates the relationship between personality and aesthetic preference, in relation to a specific category of objects (hats). An online questionnaire showing grayscale images of 34 iconic types of hats (Set 1) and eight types of baseball caps (Set 2) was presented to 539 Italian adults, asking them to rate how much they liked them and how likely they were to wear them. The Big Five Inventory-2 was used to evaluate personality. Significant associations were found between the five personality traits (open mindedness, conscientiousness, extraversion, agreeableness and negative emotionality) and the participants’ ratings of both liking and willingness to wear the hats in both Sets 1 and 2. The results of the study are relevant not only for basic research but also, potentially, suggest applicative developments in the domain of design and customized recommendation systems.

## 1. Introduction

Personality is widely recognized as important for understanding the differences in cognition and behavior between individuals (Ackerman & Heggestad, 1997; DeYoung, 2011). Its relationship with intelligence has been extensively studied in academic literature (e.g., Ackerman, 1996; Chamorro-Premuzic & Furnham, 2005; Furnham & Thorne, 2013; Matthews et al., 2006; Mussel, 2010; von Stumm & Ackerman, 2013). Of particular interest for this paper is that personality shapes creativity, learning, and decision making across both aesthetic and applied domains (DeYoung, 2011; O’Connor et al., 2022; Matthews et al., 2006; von Stumm et al., 2011; Yu et al., 2023). Our study focused on the aesthetic domain, investigating the association between personality (as described by the Big Five) and aestheticpreference for a specific kind of object: hats.

### The Interaction Between Personality and Aesthetic Preferences in Basic and Applied Psychology Research

Psychologists’ interest in human aesthetic visual preferences is not new. We know that people in general tend to value aesthetic features such as symmetry (Al-Rifaie et al., 2017; Azemati et al., 2020; Bauerly & Liu, 2008; Bertamini et al., 2019; Makin et al., 2014; Pecchinenda et al., 2014; Tyler, 2003), curvature (Chuquichambi et al., 2022; G. B. Corradi & Munar, 2020; G. Corradi et al., 2019; Gómez-Puerto et al., 2016, 2018; Palumbo et al., 2021, 2022; Sinico et al., 2021; Soranzo et al., 2024), balance (Post et al., 2016), neatly organized compositions (Van Geert & Wagemans, 2021), saturated and bright colors (Palmer et al., 2013; Palmer & Schloss, 2010).

The existence of general shared preferences does not preclude the influence of individual differences, and indeed several studies have investigated the importance of personality traits in modulating preferences. For instance, in visual art, preferences for modern or figurative art styles can be partially predicted by personality traits such as extraversion (Chamorro-Premuzic & Furnham, 2004; Furnham & Chamorro-Premuzic, 2004), negative emotionality (Furnham & Walker, 2001a), agreeableness and open mindedness (Chamorro-Premuzic et al., 2009). Appreciation of impressionist art was associated with agreeable, conscientious and less open individuals, whereas appreciation of cubism was associated with extroverts (Chamorro-Premuzic et al., 2009), and appreciation of pop art (Furnham & Walker, 2001b) and abstract art (Feist & Brady, 2004; Furnham & Walker, 2001a) with open minded people–open mindedness is also associated with higher appreciation of art in general (Feist & Brady, 2004). Clear associations were also found between personality and architectural preferences (Dehghani Tafti et al., 2024; Hartog et al., 2018), personality and music preferences (e.g., Rentfrow & Gosling, 2003), personality and design preferences (De Bont et al., 1992, Myszkowski & Storme, 2012; Yu et al., 2023); for instance, Myszkowski and Storme (2012) showed that people scoring low in open mindedness or high in agreeableness are more attracted (i.e., higher rating preferences) to visual design aspects of objects. Yu et al. (2023) found that preference for wooden furniture tends to be associated with low negative emotionality and high scores in extraversion, agreeableness and conscientiousness. De Bont et al. (1992) showed that people with a high tolerance for ambiguity (i.e., high open mindedness) have a more positive attitude towards coffee machine designs that deviate from the standard than people with lower scores on the same trait. Personality traits such as narcissistic orientation (Kang & Park, 2016), open mindedness (Fujiwara & Nagasawa, 2015a, 2015b), and extravagance (Greenberg et al., 2019) influence preferences for luxury design products. K. Chen et al. (2019) found significant associations between preferences for specific shapes, textures, and colors in elderly shoe designs and personality traits such as dominance, influence, stability, and compliance. Beyond design, but still remaining in the realm of sensory experiences, Burro et al. (2023) found a systematic and gender-independent association between personality traits and wine preferences. For example, open-minded people prefer wines with a persistent flavor and high tannin content, while they do not like sapidity; sociable people like wines with a high alcohol content and a complex bouquet; and extroverts prefer more acidic wines.

A relatively small number of studies have examined the relationship between personality and clothing preferences (see Hur et al., 2025, for a review). Clothing choices certainly respond to a criterion of adaptation to the contextual (geographical-climatic or cultural-historical) conditions in which people live (e.g., Ou et al., 2004; Palmer & Schloss, 2010). However, clothing also has an identifying function in the social relationships between people (Berger & Heath, 2007; Ratner & Kahn, 2002). The hypothesis that clothing choices are specifically linked to customers’ personalities was first introduced by Dolich (1969). He proposed the affinity link hypothesis, which posits that consumers prefer to purchase products and brands whose personalities best reflect their own. Since then, some studies have tested this hypothesis focusing on brand personalities (Huang et al., 2012; Maehle & Shneor, 2010; Mulyanegara et al., 2007; Munasinghe, 2018), product personality (e.g., Brunel & Kumar, 2007; Desmet et al., 2008; Dumitrescu, 2019; Govers & Schoormans, 2005; Matz et al., 2016), or the correspondence between a product’s perceived and intended (by the designer) personality (Govers et al., 2003; Ortíz et al., 2011). In this literature, the expression “brand personality” or “object personality” indicate anthropomorphic characteristics associated with a brand, such as being honest, sunny, charming, or tenacious (e.g., Maehle & Shneor, 2010; Malhotra, 1981; Plummer, 2000). Consumers prefer brands whose personality is congruent with their self-concept (e.g., Keller, 1993; Huang et al., 2012; Johar & Sirgy, 1991; Maehle & Shneor, 2010). For example, conscientious consumers prefer “trusted” brands, whereas extroverted consumers prefer “sociable” brands (Mulyanegara et al., 2007). If brands have their own personality, products of the same brand may differ too; for instance, it was found that customers associated Volkswagen Beetle and Volkswagen Touareg with a cheerful and friendly personality and a dominant and harsh personality, respectively, and that they tended to prefer the model that reflected their own personality (Govers, 2004; Govers & Schoormans, 2005; Mugge et al., 2009).

**Figure 1:**
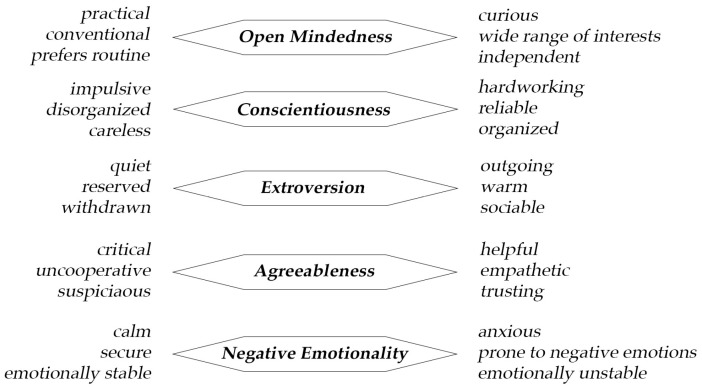
The Big Five personality traits.

In this paper, we focus on people’s personalities rather than brands or product personalities. We explore whether stable associations exist between aesthetic preferences for specific types of hats and the five traits of the Big Five personality model (McCrae & John, 1992; Goldberg, 1990), which is the most widely accepted and used personality model in current psychological literature. See Figure 1 for an outline of the five factors.

Our study relates to recent contributions to the literature that have examined the role of personality in clothing preferences. In particular, Hur et al. (2025) found that, in addition to demographic factors (gender more than age) and fashion interest, some of the Big Five personality traits played an important role in predicting preferences for certain types of clothing. For instance, extraversion was associated with a preference for activewear and sportswear and with the function of clothing as a means of looking attractive. Conversely the self-expression function (i.e., the function of emphasizing individuality) was positively predicted by open mindedness, and the concealment function (i.e., the function of hiding oneself) was positively predicted by negative emotionality and negatively predicted by extraversion.

The current study shifts the focus of the investigation from clothing preferences to hat preferences, that is, preferences for a type of clothing accessory. In Western culture, hats are not mandatory clothing items such as shoes, shirts or trousers, and in this sense hats seemed to us to be an interesting item to consider where aesthetic preference might play a decisive role. The results of our study are of interest for basic research but could inform design choices and marketing strategies. Indeed, understanding which hat models are preferred or disliked by individuals with specific personalities could guide the creation of new product designs (Prieto et al., 2019) and also assist manufacturers and retailers in implementing more effective recommendation systems, thereby enhancing the likelihood of suggesting the right product to the right individuals.

## 2. The Study

This study was designed to investigate three main aspects.

(a) *The relationship between “I like it” and “I would wear it.”* We wondered whether the hat models that people like are the same ones they would be willing to wear or whether, conversely, some differences between the two would emerge. Evidence of a positive correlation between liking and owning clothing emerged in Hur et al. (2025), but this was modulated by individual differences. In our study, we expected to find a positive correlation between responses indicating liking and willingness to wear, but also anticipated differences. This is primarily because gender likely affects the two judgements differently (namely, willingness to wear more than liking). Furthermore, personality may impact the two ratings to different extents. For instance, introverts may like hats that they would not be willing to wear, while open-minded people may be willing to wear hats they do not like as much. Investigating the relationship between liking and willingness to wear is interesting for psychological basic research reasons, but has also a methodological relevance for applicative studies. If the answers to the two questions (liking and willingness to wear) do not overlap, collecting only data about liking would provide incomplete information that could lead to biased behavioral (purchase) predictions.

(b) *The relationship between participants’ personality traits and their hat preferences*. We wondered whether there was a relationship between the five personality factors of the Big Five model and the positive or negative aesthetic response to and/or willingness to wear specific types of hats. This was the fundamental question of our study. We expected relationships to emerge for at least some, if not all, of the traits. However, we were unable to predict the specific items involved in each association.

(c) *The possibility of using personality traits to predict both “macro-preferences” and “micro-preferences.”* The stimuli presented to participants in the current study included 34 iconic hats (i.e., hats that are well known in the contemporary Western world, covering a wide range of models) and eight variants of the baseball hat. This choice was influenced by our interest in testing the level at which customers’ personality can be used to predict their preferences. The aesthetic and functional differences between the baseball cap models were smaller than those between the 34 iconic hats. Therefore, we expected different preference profiles based on personality traits to emerge more clearly in the wider, more variable set of hats (Set 1) than in the subset of baseball caps (Set 2).

### 2.1. Method

#### 2.1.1. Participants

Five hundred thirty-nine adults voluntarily took part in the study and completed it (396 females, 143 males; mean age = 24.799; standard deviation = 11.001). Participants were recruited at the Universities of Macerata and Verona, and through social media (Facebook, WhatsApp, and Instagram). The study conforms to the ethical principles of the Declaration of Helsinki (World Medical Association, 2013), and ethical approval was obtained from the University of Macerata (C.E. 17/3/2023).

#### 2.1.2. Materials

An online questionnaire in Italian was used for data collection. It comprised four sections (the Personal Information, Personality Test, Iconic Hats, and Baseball Hats sections), preceded by an Informed Consent section.

In the first section, personal information was collected (gender, age).

In the Personality Test section, the 60 items forming the Big Five Inventory-2 (Soto & John, 2017) were used, in the Italian version (Burro et al., 2025). The participants had to rate the extent to which they agreed or disagreed with a statement as descriptive of their own personality, using a five-point scale (strongly disagree, slightly disagree, neither agree nor disagree, slightly agree, and strongly agree).

**Figure 2:**
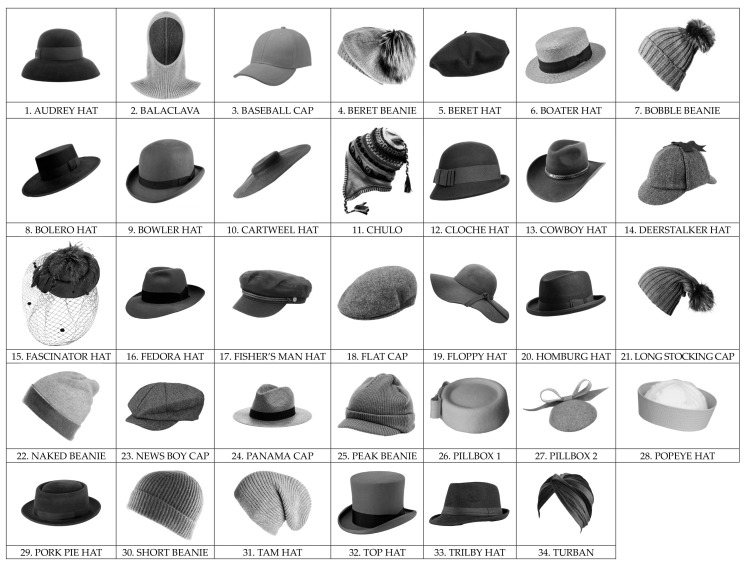
The 34 iconic hat models (set 1) used in the study.

In the Iconic Hats section, 34 grayscale images (930 × 540 pixels) were presented, one at a time (the full list of hats and their names is shown in Figure 2). They were followed by two continuous 0–100 scales, which participants used to rate how much they liked the displayed hat (preference) and how much they were willing to wear it (willingness to wear). In each image, the hat was displayed in a spatial orientation that allowed participants to notice all the typical features defining the shape of the hat.

**Figure 3:**
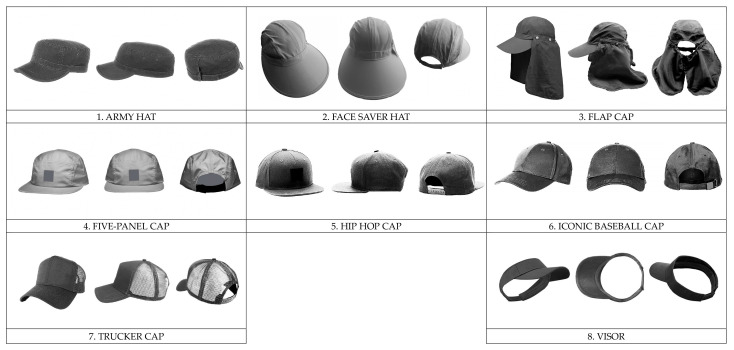
The 8 baseball cap models (set 2) used in the study.

In the Baseball Hats section, eight models of baseball hats were shown, one at a time (the hats and their names are shown in Figure 3), and the participants rated them following the same two scales (preference and willingness to wear) used for the bigger sample of iconic hats. The only difference was that each hat was shown from three different angles in this case, to enable participants to observe the distinctive features of the models presented, given that baseball caps are a specific type of hat and are therefore quite similar in terms of their overall shape. The size of the images was 930 × 540 pixels.

Both iconic and baseball caps models were selected based on an online search, focusing on those that frequently appeared within at least one of two major retail sites for clothing and fashion accessories—one primarily serving the Italian market (Zalando, 2025a, 2025b) and the other primarily serving the North American market (MyStyleBox, 2023). “Iconic,” when referring to a hat model (or any other fashion or design object), means that it is extremely recognizable, symbolic, and perfectly represents an era, style, or culture, becoming a strong and memorable image that embodies an entire concept, such as the Borsalino hat representing classic elegance or the cowboy hat representing the American West. It is not only beautiful, but also an emblem of something easily associated with it and enduring over time. The models selected for both sets of stimuli used in the study (i.e., iconic hats and baseball caps) were approved by an expert designer as being representative. Brands were removed from the images. Color information was also removed, and all images were in grayscale. We are aware that color can have a significant impact on decision making, causing us to like a hat purely because of its color, despite not liking its shape. However, we were interested in the shape of the hats, so we removed the color to be able to focus on shape.

#### 2.1.3. Procedure

The participants accessed the questionnaire from their computers, tablets, or smartphones, using the link shared by the researchers via social media and among the students and staff of the University of Verona and Macerata. An initial page presented the aims and contents of the study (the Informed Consent Form). Participants who agreed to participate accessed the first section of the questionnaire, where their personal information was collected. The next three sections (Personality, Iconic Hats, and Baseball Hats) followed in random order. The order of the items within each section was also randomized between participants.

No time limits were set. Participants could observe each stimulus (hat image) for as long as they liked before giving their ratings. The questionnaire could either be completed all at once in one session or after several breaks (participants only had to leave the browser open; they could resume the compilation whenever they wished). The time needed to complete the questionnaire was 25 min on average.

#### 2.1.4. Data Analysis

All analyses were conducted using the statistical software R (Version 4.5.2, R Core Team, 2025). To examine the strength and direction of the association between participants’ responses concerning liking and willingness to wear, r Pearson’s correlation analysis was used (corrtable R-package, Version 0.1.1; van der Laken, 2023).

We used exploratory structural equation modeling (ESEM) (lavaan R-package: Rosseel, 2012) to identify factor structures underlying our data, for sets 1 and 2 separately. ESEM integrates exploratory and confirmatory modeling within a single framework and allowed us to avoid loss of statistical power due to sample splitting (Asparouhov and Muthén, 2009). We then used structural equation modeling (SEM) to study the effect of gender and personality on the emerging factors. The number of factors was determined using multiple empirical criteria, including the two most commonly used: parallel analysis and inspection of the scree plot (parameters R-package: Lüdecke et al., 2020).

To examine gender differences in these associations, measurement invariance across gender was tested prior to conducting multi-group SEM (Byrne et al., 1989). Invariance decisions were based on changes in fit indices (|ΔCFI| ≤ 0.010; |ΔRMSEA| ≤ 0.015; |ΔSRMR| ≤ 0.010; F. F. Chen, 2007) The model was estimated using robust maximum likelihood (MLR). Model fit was evaluated using multiple indices (CFI ≥ 0.90; TLI ≥ 0.90; RMSEA ≤ 0.08; SRMR ≤ 0.08; Kline, 2016).

RMSEA-based post hoc power analysis (MacCallum et al., 1996) was conducted to evaluate the adequacy of the sample size for testing global model fit (semPower, Moshagen & Bader, 2024).

## 3. Results

The following subsections describe the results for the larger set of iconic hats (Section 3.1) and the smaller set of baseball hats (Section 3.2). First, we examined the relationship between liking and willingness to wear (see Section 3.1.1 and Section 3.2.1). Next, we analyzed the underlying factor structure of our data (see Section 3.1.2, Section 3.1.4, Section 3.2.2 and Section 3.2.4). Finally, we investigated whether significant associations emerged between personality traits and preferences, and whether these were influenced by gender, with respect to both liking (Section 3.1.3 and Section 3.2.3) and willingness to wear (Section 3.1.5 and Section 3.2.5).

### 3.1. The Larger Set: Various Types of Iconic Hats

#### 3.1.1. The Relationships Between “I Like It” and “I Would Wear It” (Set 1)

As shown in Table 1, there was a significant correlation between the two ratings for each hat model. However, these correlations were far from perfect (the range of Pearson’s r varied from 0.605 to 0.856). For this reason, we analyzed the two ratings separately in all subsequent analyses.

#### 3.1.2. The Factors Underlying Responses of Liking (Set 1)

**Figure 4:**
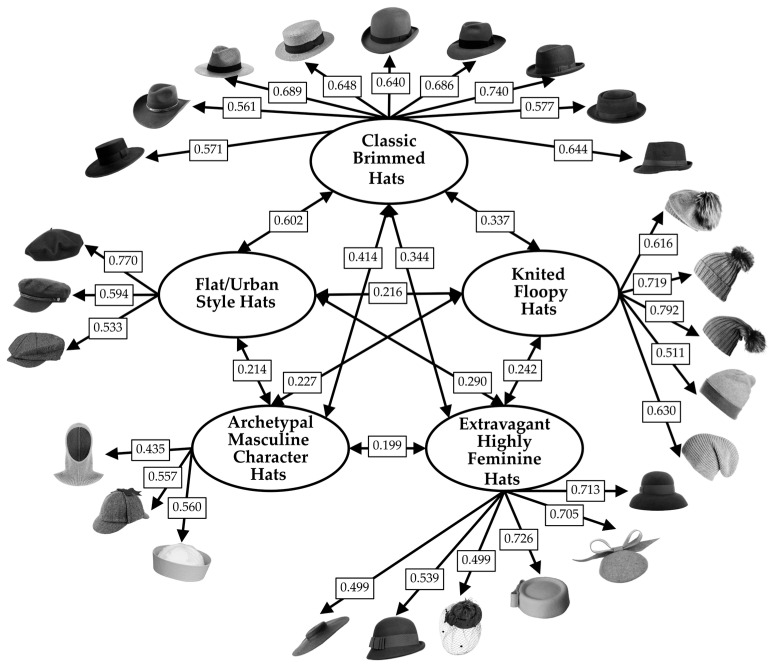
Standardized factor loadings (λ) of the 34 iconic hats in the five factors emerged from the ESEM conducted on liking, for the set of iconic hats (set 1). Note. Only loadings ≥ |0.30| and that do not show cross-loadings (threshold equal to 0.2) are shown.

The first ESEM was conducted to identify common factors underlying responses of liking given by participants to the set of iconic models. Preliminary analyses indicated that the data were suitable for factor analysis (Kaiser–Meyer–Olkin measure of sampling adequacy = 0.929; Bartlett’s test of sphericity, χ^2^(561) = 9030.057, *p* < 0.001). The number of factors was determined using a combination of empirical criteria, which included parallel analysis and an inspection of the scree plot. These methods supported the five-factor solution shown in Figure 4. A five-factor ESEM was estimated using robust maximum likelihood estimation (MLR) with geomin rotation. The five-factor ESEM demonstrated adequate model fit, χ^2^(401) = 1113.828, *p* < 0.001, CFI = 0.918, TLI = 0.895, RMSEA = 0.057, 90% CI [0.053, 0.061], SRMR = 0.031. Standardized factor loadings are reported in Figure 4.

Factor 1 was characterized by high loadings on hats that are flat-topped (beret hat, fisherman hat, news boy hat) and was labeled “Flat/Urban Style Hats”.

Factor 2 was characterized by high loadings on unstructured hats (beret beanie, bobble beanie, long stoking, naked beanie, tam hat) and was labeled “Knitted Floopy Hats”.

Factor 3 was characterized by high loadings on highly feminine hats (cartwheel hat, cloche hat, fascinator hat, pillbox 1, pillbox 2, Audrey hat) and was labeled “Extravagant Highly Feminine Hats”.

Factor 4 was characterized by high loadings on hats associated with archetypal characters from cinema or society (balaclava, deerstalker hat, Popeye hat) and was labeled “Archetypal Masculine Character Hats”.

Factor 5 was characterized by high loadings on classic hats with wide brim that goes all the way around (bolero hat, cowboy hat, Panama hat, boater hat, bowler hat, fedora hat, homburg hat, pork pie hat, trilby hat) and was labeled “Classic Brimmed Hats”.

Latent factor correlations ranged from 0.199 to 0.602 (mean = 0.308), indicating related but distinct dimensions of hat preferences. McDonald’s ω reliability was adequate for all factors (F1 = 0.701; F2 = 0.807; F3 = 0.859; F4 = 0.699; F5 = 0.913)

#### 3.1.3. The Effect of Personality Traits and Gender on Liking (Set 1)

Following the ESEM, a structural equation model (SEM) was used to examine the extent to which the Big Five personality traits predicted the five hat-preference factors. For SEM, each hat item was assigned to the factor on which it showed the highest standardized loading in the ESEM.

The five hat-preference factors were specified as latent outcome variables, each regressed on the five Big Five personality traits. To examine gender differences in these associations, measurement invariance across gender was tested prior to conducting multi-group SEM (Byrne et al., 1989). After establishing metric invariance (ΔCFI = 0.003; ΔRMSEA = 0.000; ΔSRMR = 0.004), structural paths were compared between males and females. Constraining the structural paths to equality across gender did not result in a meaningful decrease in model fit (ΔCFI = 0.001; ΔRMSEA = 0.001; ΔSRMR = 0.003), suggesting comparable associations across males and females. For these reasons the more parsimonious single-group model with common regression paths across males and females was retained for interpretation.

The model was estimated using robust maximum likelihood (MLR). The SEM showed the following fit to the data: χ^2^(393) = 956.605, *p* < 0.001, CFI = 0.902, TLI = 0.897, RMSEA = 0.055, 90% CI [0.051, 0.60], SRMR = 0.051. Although TLI was below conventional cutoff for excellent fit, this value is not uncommon in complex SEMs with many indicators and soft psychological constructs. Other fit indices indicated an acceptable overall fit.

**Figure A1:**
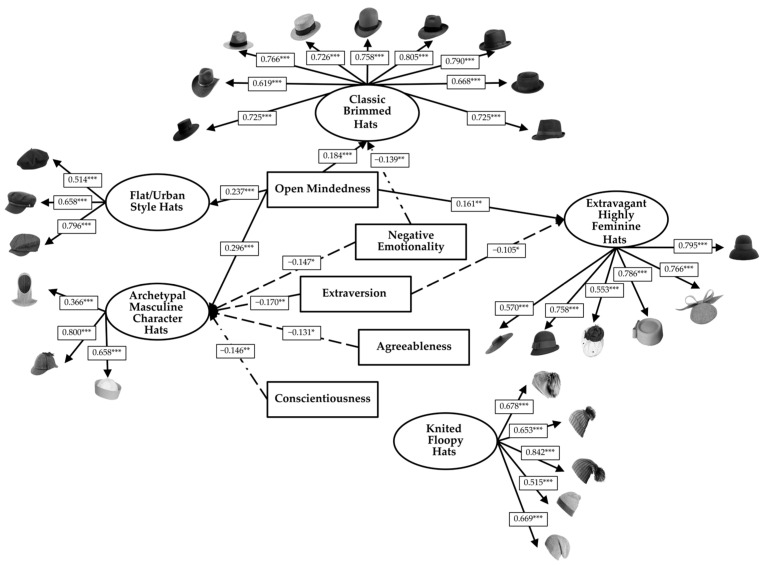
Diagram plots of the structural equation model (SEM) conducted on the five factors that emerged from the exploratory structural equation modellings (ESEMs) on set 1. The plot refers to ratings of liking. For reasons of plot readability, we have shown only significant β and λ values in the image. Note. Standardized regression coefficients (β and λ) are reported as values on the arrows. Significance Codes: *p* < 0.05 (*); *p* < 0.01 (**); *p* < 0.001 (***). For β values, negative arrows are dashed and positive arrows are solid.

An RMSEA-based post hoc power analysis was conducted to evaluate the adequacy of the sample size for testing global model fit. Under the alternative hypothesis of a population RMSEA of 0.08, the analysis indicated adequate power at N = 539 with 393 degrees of freedom (α = 0.05). Standardized regression coefficients (β) are reported in Table 2. The complete model’s diagram plot can be found in Appendix A, Figure A1.

Extraversion negatively predicted preference for F3-Extravagant Highly Feminine Hats (β = −0.105, *p* = 0.040) and negatively predicted preference for F4-Archetypal Masculine Character Hats (β = −0.170, *p* = 0.004).

Agreeableness negatively predicted preference for F4-Archetypal Masculine Character Hats (β = −0.131, *p* = 0.013).

Conscientiousness negatively predicted preference for F4-Archetypal Masculine Character Hats (β = −0.146, *p* = 0.006).

Negative Emotionality negatively predicted preference for F4-Archetypal Masculine Character Hats (β = −0.147, *p* = 0.011) and negatively predicted preference for F5-Classic Brimmed Hats (β = −0.139, *p* = 0.005).

Open Mindedness positively predicted preference for F1-Flat/Urban Style Hats (β = 0.237, *p* < 0.001), positively predicted preference for F3-Extravagant Highly Feminine Hats (β = 0.161, *p* = 0.001), positively predicted preference for F4-Archetypal Masculine Character Hats (β = 0.296, *p* < 0.001) and positively predicted preference for F5-Classic Brimmed Hats (β = 0.184, *p* < 0.001).

#### 3.1.4. The Factors Underlying Responses of Willingness to Wear (Set 1)

**Figure 5:**
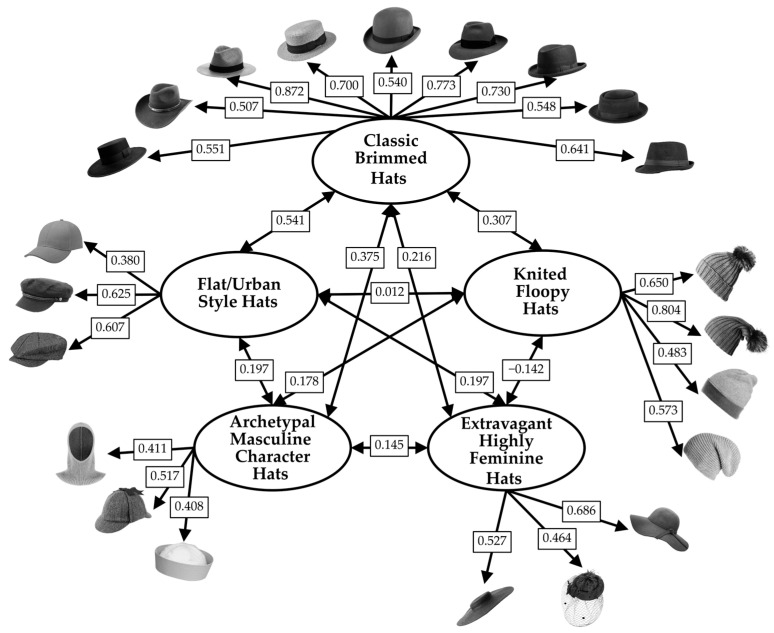
Standardized factor loadings (λ) of the 34 iconic hats in the five factors emerged from the ESEM conducted on willingness to wear, for the set of iconic hats (set 1). Note. Only loadings ≥ |0.30| and that do not show cross-loadings (threshold equal to 0.2) are shown.

Preliminary analyses indicated that the data were suitable for factor analysis (Kaiser–Meyer–Olkin measure of sampling adequacy = 0.909; Bartlett’s test of sphericity, χ^2^(561) = 7652.555, *p* < 0.001). An ESEM was conducted to identify common factors underlying responses of willingness to wear. As for the previous ESEM, the number of factors was determined using multiple criteria, including the parallel analysis and inspection of the scree plot. A five-factor ESEM was estimated using robust maximum likelihood estimation (MLR) with geomin rotation. The five-factor ESEM demonstrated adequate model fit, χ^2^(401) = 1044.280, *p* < 0.001, CFI = 0.937, TLI = 0.912, RMSEA = 0.048, 90% CI [0.042, 0.054], SRMR = 0.032. Standardized factor loadings are reported in Figure 5.

Factor 1 was characterized by high loadings on urban hats (baseball cap, fisherman hat, news boy hat) and was labeled “Flat/Urban style Hats”.

Factor 2 was characterized by high loadings on unstructured hats (bobble beanie, long stoking, naked beanie, tam hat) and was labeled “Knitted Floopy Hats”.

Factor 3 was characterized by high loadings on highly feminine hats (cartwheel hat, fascinator hat, and floopy hat) and was labeled “Extravagant Highly Feminine Hats”.

Factor 4 was defined by high loadings on hats associated with archetypal characters from cinema or society (balaclava, deerstalker hat, Popeye hat) and was therefore labeled “Archetypal Masculine Character Hats”.

Factor 5 was characterized by high loadings on typical hats with wide brim that goes all the way around (bolero hat, cowboy hat, Panama hat, boater hat, bowler hat, fedora hat, homburg hat, pork pie hat, trilby hat) and was labeled “Classic Brimmed Hats”.

Latent factor correlations ranged from 0.012 to 0.541 (mean = 0.220), indicating related but distinct dimensions of hat preferences. McDonald’s ω reliability was adequate for all factors (F1 = 0.664; F2 = 0.685; F3 = 0.742; F4 = 0.672; F5 = 0.897).

The five factors essentially correspond to the typologies that emerged for liking. The hats that contribute to Factors 4 and 5 are exactly the same, while there are differences regarding some hats for the other factors. These differences will be discussed in the final section.

#### 3.1.5. The Effect of Personality Traits and Gender on Willingness to Wear (Set 1)

As we already did for liking, also in this case a SEM was used to examine the extent to which the Big Five personality traits predicted the five hat-preference factors. Each hat item was assigned to the factor on which it showed the highest standardized loading in the ESEM.

The five hat-preference factors were specified as latent outcome variables, each regressed on the five Big Five personality traits. To examine gender differences in these associations, measurement invariance across gender was tested prior to conducting multi-group SEM. After establishing metric invariance (ΔCFI = 0.006; ΔRMSEA = 0.001; ΔSRMR = 0.003), structural paths were compared between males and females. Similar to what was found for liking, constraining the structural paths to equality across gender did not result in a meaningful decrease in model fit (ΔCFI = 0.002; ΔRMSEA = 0.001; ΔSRMR = 0.004), suggesting comparable associations across males and females. For these reasons the more parsimonious single-group model with common regression paths across males and females was retained for interpretation.

The model was estimated using robust maximum likelihood (MLR). The SEM showed this fit to the data, χ^2^(280) = 871.001, *p* < 0.001, CFI = 0.891, TLI = 0.875, RMSEA = 0.052, 90% CI [0.048, 0.56], SRMR = 0.058. Although the CFI and TLI were below conventional cutoffs for excellent fit, these values are not uncommon in complex SEMs with many indicators and soft psychological constructs. Other fit indices indicated an acceptable overall fit.

**Figure A2:**
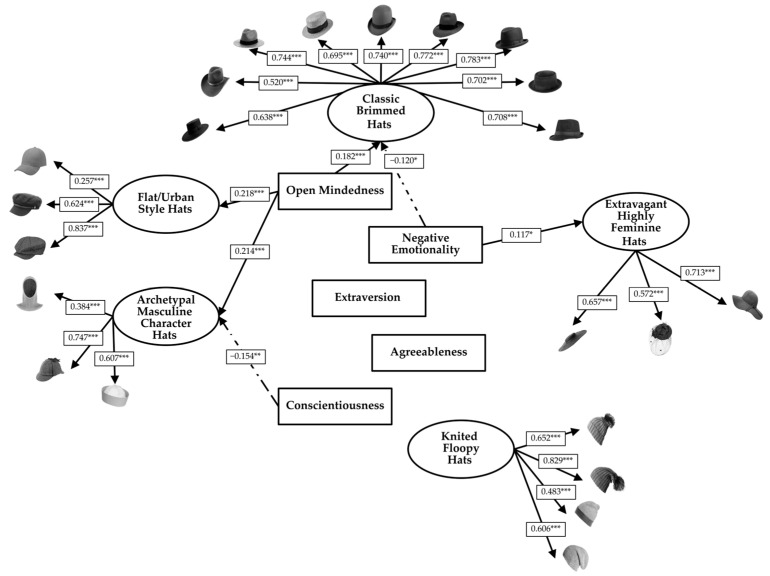
Diagram plots of the structural equation model (SEM) conducted on the five factors that emerged from the exploratory structural equation modellings (ESEMs) on set 1. The plot refers to ratings of willingness to wear. For reasons of plot readability, we have shown only significant β and λ values in the image. Note. Standardized regression coefficients (β and λ) are reported as values on the arrows. Significance Codes: *p* < 0.05 (*); *p* < 0.01 (**); *p* < 0.001 (***). For β values, negative arrows are dashed and positive arrows are solid.

An RMSEA-based post hoc power analysis was conducted to evaluate the adequacy of the sample size for testing global model fit. Under the alternative hypothesis of a population RMSEA of 0.08, the analysis indicated adequate power at N = 539 with 280 degrees of freedom (α = 0.05). Standardized regression coefficients (β) are reported in Table 3. The complete model’s diagram plot can be found in Appendix A, Figure A2.

No association was found between two traits, extraversion and agreeableness, and any of the five factors that emerged from the ESEM.

Conscientiousness was found to negatively predict willingness to wear F4-Archetypal Masculine Character Hats (β = −0.154, *p* = 0.002). This is consistent with the results for liking. The more conscientious someone is, the less they like these hats and the less willing they are to wear them.

Negative emotionality predicted unwillingness to wear F5-Classic Brimmed Hats (β = −0.120, *p* = 0.011), while it positively predicted preference for F3-Extravagant Highly Feminine Hats (β = 0.117, *p* = 0.027). This means that the more emotionally unstable someone is, the less they like the idea of wearing brimmed hats, and the more they like the idea of wearing extravagant, highly feminine hats. The opposite is true for emotionally stable people.

Open mindedness was found to positively predict the willingness to wear F1-Flat/Urban-Style Hats (β = 0.218, *p* < 0.001), F4-Archetypal Masculine Character Hats (β = 0.214, *p* < 0.001), and F5-Classic Brimmed Hats (β = 0.182, *p* < 0.001). These results are consistent with liking preferences. An exception is represented by F3-Extravagant Highly Feminine Hats, which open-minded people liked, but were not willing to wear.

### 3.2. Baseball Caps

#### 3.2.1. Relationships Between “I Like It” and “I Would Wear It” (Set 2)

As in the iconic hats, we first explored the relationship between liking and willingness to wear (see Table 4). The responses were highly correlated for all tested models, but not perfectly so (the range of Pearson’s r varied from 0.612 to 0.867).

#### 3.2.2. The Factors Underlying Responses of Liking (Set 2)

**Figure 6:**
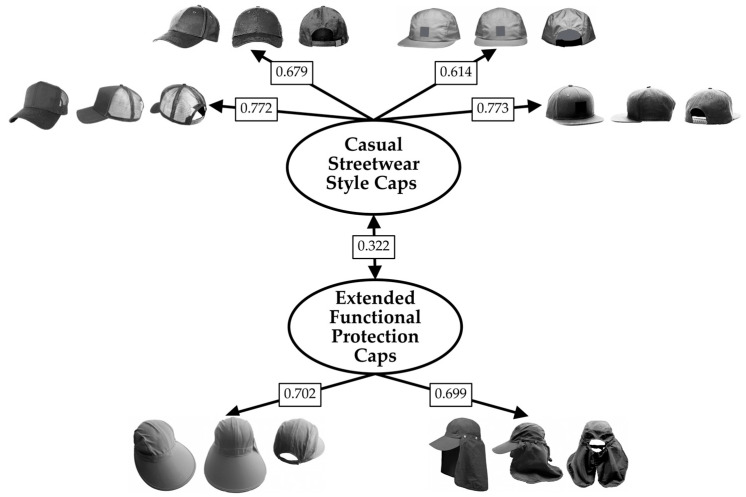
Standardized factor loadings (λ) of the 8 baseball caps (set 2) in the two factors emerged from the ESEM conducted on liking. Note. Only loadings ≥ |0.30| and that do not show cross-loadings (threshold equal to 0.2) are shown.

Preliminary analyses indicated that the data were suitable for factor analysis (Kaiser–Meyer–Olkin measure of sampling adequacy = 0.799; Bartlett’s test of sphericity, χ^2^(28) = 1138.238, *p* < 0.001). The number of factors was determined using multiple empirical criteria, including parallel analysis and inspection of the scree plot. Both methods supported a two-factor solution. A two-factor ESEM was estimated using robust maximum likelihood estimation (MLR) with geomin rotation. The two-factor ESEM demonstrated adequate model fit, χ^2^(13) = 54.356, *p* < 0.001, CFI = 0.967, TLI = 0.929, RMSEA = 0.073, 90% CI [0.053, 0.094], SRMR = 0.026. Standardized factor loadings are reported in Figure 6.

Factor 1 was characterized by high loadings on coverage-oriented protection caps (face saver hat, flap cap) and was labeled “Extended Functional Protection Caps”. Factor 2 was characterized by high loadings on everyday baseball caps (trucker, baseball iconic, five panel, hip hop cap) and was labeled “Casual Streetwear Style Caps”.

Latent factor correlation was equal to 0.322, indicating related but distinct dimensions of hat preferences. McDonald’s ω reliability was adequate for all factors (F1 = 0.658; F2 = 0.811).

#### 3.2.3. The Effect of Personality Traits and Gender on Liking (Set 2)

A SEM was used to examine the extent to which the Big Five personality traits predicted the two factors underlying preference ratings. Each hat item was assigned to the factor on which it showed the highest standardized loading in the ESEM.

The two hat-preference factors were specified as latent outcome variables, each regressed on the five Big Five personality traits. To examine gender differences in these associations, measurement invariance across gender was tested prior to conducting multi-group SEM. After establishing metric invariance (ΔCFI = 0.000; ΔRMSEA = 0.002; ΔSRMR = 0.003), structural paths were compared between males and females. Constraining the structural paths to equality across gender did not result in a meaningful decrease in model fit (ΔCFI = 0.004; ΔRMSEA = 0.003; ΔSRMR = 0.006), suggesting comparable associations across males and females. For these reasons the more parsimonious single-group model with common regression paths across males and females was retained for interpretation. The model was estimated using robust maximum likelihood (MLR). The SEM showed the following fit to the data, χ^2^(28) = 90.215, *p* < 0.001, CFI = 0.935, TLI = 0.895, RMSEA = 0.065, 90% CI [0.050, 0.080], SRMR = 0.036. Although the TLI was below conventional cutoff for excellent fit, other fit indices indicated an acceptable overall fit.

**Figure A3:**
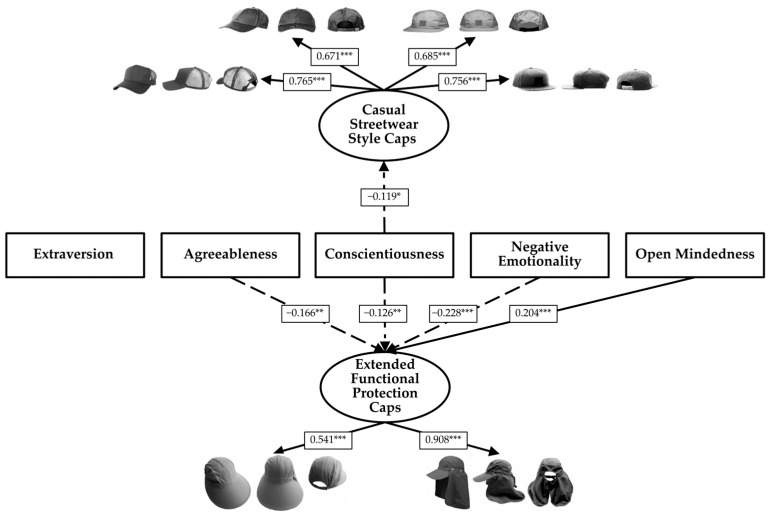
Diagram plots of the structural equation models (SEM) conducted on the two factors that emerged from the exploratory structural equation modeling (ESEM) on set 2. The plot refers to ratings of liking. For reasons of plot readability, we have shown only significant β and λ values in the image. Note. Standardized regression coefficients (β and λ) are reported as values on the arrows. Significance Codes: *p* < 0.05 (*); *p* < 0.01 (**); *p* < 0.001 (***). For β values, negative arrows are dashed and positive arrows are solid.

An RMSEA-based post hoc power analysis was conducted to evaluate the adequacy of the sample size for testing global model fit. Under the alternative hypothesis of a population RMSEA of 0.08, the analysis indicated adequate power at N = 539 with 28 degrees of freedom (α = 0.05). Standardized regression coefficients (β) are reported in Table 5. The complete model’s diagram plot can be found in Appendix A, Figure A3.

Agreeableness negatively predicted preference for F1-Extended Functional Protection caps (β = −0.166, *p* = 0.001).

Conscientiousness negatively predicted preference for F1-Extended Functional Protection Caps (β = −0.126, *p* = 0.009) and negatively predicted preference for F2-Casual Streetwear Style Caps (β = −0.119, *p* = 0.026).

Negative emotionality negatively predicted preference for F1-Extended Functional Protection Caps (β = −0.228, *p* < 0.001).

Open mindedness positively predicted preference for F1-Extended Functional Protection Caps (β = 0.204, *p* < 0.001).

#### 3.2.4. The Factors Underlying Responses of Willingness to Wear (Set 2)

**Figure 7:**
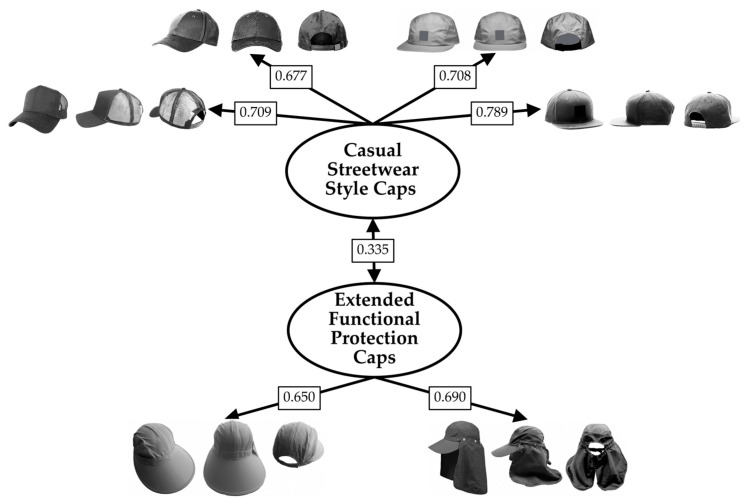
Standardized factor loadings (λ) of the 8 baseball caps (set 2) in the two factors emerged from the ESEM conducted on willingness to wear. Note. Only loadings ≥ |0.30| and that do not show cross-loadings (threshold equal to 0.2) are shown.

Preliminary analyses indicated that the data were suitable for factor analysis (Kaiser–Meyer–Olkin measure of sampling adequacy = 0.785; Bartlett’s test of sphericity, χ^2^(28) = 1069.964, *p* < 0.001). The number of factors was determined using parallel analysis and inspection of the scree plot. Both methods supported a two-factor solution. A two-factor ESEM was estimated using robust maximum likelihood estimation (MLR) with geomin rotation. The two-factor ESEM demonstrated adequate model fit, χ^2^(13) = 72.189, *p* < 0.001, CFI = 0.948, TLI = 0.897, RMSEA = 0.078, 90% CI [0.053, 0.105], SRMR = 0.032. Standardized factor loadings are reported in Figure 7. Also in this case, although the TLI was below conventional cutoff for excellent fit, the other fit indices indicated an acceptable overall fit.

As for liking (Section 3.2.2), Factor 1 was characterized by high loadings on coverage-oriented protection caps (Face Saver Hat, flap cap) and was labeled “Extended Functional Protection Caps”. Factor 2 was characterized by high loadings on everyday caps (trucker, baseball iconic, five panel, hip hop cap) and was labeled “Casual Streetwear Style Caps”.

Latent factor correlation was equal to 0.335, indicating related but distinct dimensions of hat preferences. McDonald’s ω reliability was adequate for all factors (F1 = 0.679; F2 = 0.802).

#### 3.2.5. The Effect of Personality Traits and Gender on Willingness to Wear (Set 2)

A SEM was used to examine the extent to which the Big Five personality traits predicted the two factors underlying willingness to wear ratings. For SEM, each hat item was assigned to the factor on which it showed the highest standardized loading in the ESEM.

The two factors were specified as latent outcome variables, each regressed on the five Big Five personality traits. To examine gender differences in these associations, measurement invariance across gender was tested prior to conducting multi-group SEM. After establishing metric invariance (ΔCFI = 0.007; ΔRMSEA = 0.000; ΔSRMR = 0.005), structural paths were compared between males and females. Constraining the structural paths to equality across gender in this case did result in a meaningful decrease in model fit (ΔCFI = 0.012; ΔRMSEA = 0.001; ΔSRMR = 0.008), suggesting not comparable associations across males and females. For this reason subsequent analyses were conducted using the multi-group model with freely estimated structural paths. The model was estimated using robust maximum likelihood (MLR). The SEM showed this fit to the data, χ^2^(28) = 127.645, *p* < 0.001, CFI = 0.910, TLI = 0.885, RMSEA = 0.066, 90% CI [0.052, 0.081], SRMR = 0.039. Also in this case, although the TLI was below conventional cutoff for excellent fit, the other fit indices indicated an acceptable overall fit.

**Figure A4:**
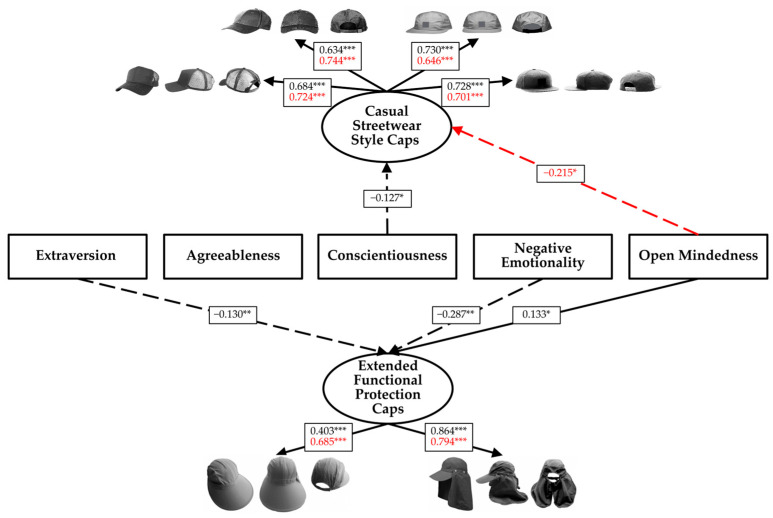
Diagram plots of the structural equation model (SEM) conducted on the two factors that emerged from the exploratory structural equation modeling (ESEM) on set 2. The plot refers to ratings of willingness to wear. For reasons of plot readability, we have shown only significant β and λ values in the image. Note. Standardized regression coefficients (β and λ) are reported as values on the arrows. Significance Codes: *p* < 0.05 (*); *p* < 0.01 (**); *p* < 0.001 (***). For β values, negative arrows are dashed and positive arrows are solid. The red color refers to males, black to females.

An RMSEA-based post hoc power analysis was conducted to evaluate the adequacy of the sample size for testing global model fit. Under the alternative hypothesis of a population RMSEA of 0.08, the analysis indicated adequate power at N = 539 with 28 degrees of freedom (α = 0.05). Standardized regression coefficients (β) are reported in Table 6. The complete model’s diagram plots can be found in Appendix A, Figure A4.

For male participants, open mindedness negatively predicted preference for F2-Casual Streetwear Style Caps (β = −0.215, *p* = 0.030).

For female participants, open mindedness positively predicted preference for F1-Extended Functional Protection Caps (β = 0.133, *p* = 0.049). Extraversion, negatively predicted preference for F1-Extended Functional Protection Caps (β = −0.130, *p* = 0.032). The same negative association was found for negative emotionality and preference for F1-Extended Functional Protection Caps (β = −0.287, *p* = 0.001). A negative association was also found for conscientiousness and F2-Casual Streetwear Style Caps (β = −0.127, *p* = 0.031).

## 4. Discussion

As numerous studies have indicated, personality appears to be an important human factor in predicting preferences across various domains (Ozer & Benet-Martinez, 2006). This paper contributes to an existing but underdeveloped line of research (briefly reviewed in the introduction to this paper) that investigates whether people’s personality traits predict preferences for specific types of products. The focus was on hats as a special type of clothing item. The results of our study support the hypothesis that there is a relationship between personality and hat preference. In this sense, our findings contribute to the existing body of research on clothing preferences (e.g., Hur et al., 2023, 2025; Jegethesan et al., 2012; Stolovy, 2021), extending the items considered (hats in our case) and the populations used, that is, from the UK, USA, Australian and Israeli populations (respectively considered in the previously mentioned studies), to the Italian population.

Specifically, our main aim was to study whether hat preference could be predicted by personality traits, and if so, to what extent these associations differed between males and females, or whether they were gender independent. This was examined at two levels: ‘macro’, in relation to a wide range of iconic hat designs (Figure 2), and ‘micro’, with respect to baseball caps (Figure 3). Preference was studied in terms of both liking and willingness to wear. A secondary aim of the study was to examine the relationship between these two ratings, similar to Hur et al.’s (2025) study of clothing preferences.

The two preference ratings used in this study—liking and willingness to wear—were found to be strongly positively correlated (see Table 1 for iconic hats, and Table 4 for baseball caps). This was also reflected in the finding that the factor structure underlying the two sets of data was very similar, for both iconic hats (set 1) and baseball caps (set 2).

With respect to iconic hats, five “corresponding” factors emerged from the ESEM conducted to study liking and the ESEM conducted to study willingness to wear. These factors were labeled as follows: Flat/Urban Style Hats (Factor 1), Knitted Floppy Hats (Factor 2), Extravagant Highly Feminine Hats (Factor 3), Archetypal Masculine Character Hats (Factor 4) and Classic Brimmed Hats (Factor 5).

The hats contributing to Factors 4 (Archetypal Masculine Character Hats) and 5 (Classic Brimmed Hats) were the same in both ESEMs. However, some differences were observed for the other factors. Specifically, the beret hat disappeared from Factor 1 (Flat/Urban Style Hats) while the baseball cap appeared when willingness to wear rather than liking was analyzed. This result may be related to the age group of our participants, who were young Italians. Both the beret hat and the baseball cap are urban hats, but the former may appear more old-fashioned, while the latter is more typically associated with a young style.

In Factor 2 (Knitted Floppy Hats), willingness to wear was limited to elongated hats, with or without a tuft. The non-elongated beret beanie disappeared from the set of hats loading on this factor. This selective preference for wearing only the elongated versions of knitted floopy hats may reflect a fashion trend.

With respect to Factor 3 (Extravagant Highly Feminine Hats), willingness to wear was limited to a subset of the liked hats: the fascinator (a surprising result), the cartwheel hat and extended to another hat with a wide brim (i.e., the floppy hat), which was not among the liked hats. The two pillbox hats, the cloche hat and the Audrey hat, which contributed to Factor 2 in terms of liking, did not load onto this factor when willingness to wear was considered.

With regard to the set of baseball hats (set 2), the factors extracted from the liking and willingness to wear ratings were found to be perfectly matched. Two factors emerged in both cases. Factor 1 grouped the two more unusual baseball cap models in our set, characterized by enlarged protective features, and was named Extended Functional Protection Caps (i.e., face saver hat and flap cap). Factor 2 grouped the four most common baseball cap models (i.e., trucker cap, iconic baseball cap, five-panel cap and hip-hop cap) and was labeled Casual Streetwear Style Caps.

Turning now to the main aim of the study, which was to investigate associations between the Big Five personality traits and preference, we found significant associations for most of the traits, both when liking and willingness to wear were considered. The associations found were comparable for iconic hats (set 1) across males and females, highlighting the generalizability of these associations across gender. However, some gender differences emerged in the association between factor and personality for the baseball caps (set 2). Note that, to examine whether the structural relations between hat-preference factors and personality traits differed by gender, we used multigroup structural equation models (SEMs). These analyses address potential gender differences in the associations between variables, rather than differences in their mean levels. Therefore, the absence of gender differences in regression coefficients should not be interpreted as evidence that males and females exhibit similar average levels of liking or willingness to wear specific types of hats. These analyses do not test for gender differences in overall liking or willingness to wear certain hats; rather, they assess whether personality traits are related to hat-preference factors in a comparable manner across genders.

We will start with the iconic hats (set 1). Significant associations were found for liking and willingness to wear, with four and three traits respectively.

Open-minded individuals liked a wide range of hats with very different characteristics, ranging from classic structured hats with an upstanding crown (Factor 5-Classic Brimmed Hats) to flat hats with no crown at all, but that on the contrary sit horizontally on top of the head (Factor 1-Flat/Urban Style Hats). Likewise, they liked Extravagant Highly Feminine Hats (Factor 3) and also the Archetypal Masculine Character Hats considered in our study (Factor 4). The latter are not only visually extremely different from one another, but also strongly associated with cinema or social figures: the Popeye hat, which is iconic in American pop culture and represents working-class heroism (Popeye is a strong-willed and stubborn sailor who eats spinach and is loyal to his girlfriend, Olivia); the deerstalker hat, originally designed for Scottish deer hunters, which became famously associated with Sherlock Holmes; and the balaclava hat, associated with outdoor activities, as well as historically with military and police special forces, but at the same time also criminality. Open-minded people liked and were willing to wear all of these types of hats, with the exception of those in Factor 3-Extravagant Highly Feminine Hats. They liked these hats, but Open Mindedness does not predict willingness to wear them.

Factor 4-Archetypal Masculine Character Hats was significantly associated also with the other four traits of the Big Five: extraversion (negative association for liking), agreeableness (negative association for liking), conscientiousness (negative association for both liking and willingness to wear), and negative emotionality (negative association for liking). In other words, these hats were popular with people who were quiet and reserved (in terms of the extraversion trait), suspicious and critical (in terms of the agreeableness trait), impulsive and disorganized (in terms of the conscientiousness trait), and calm and secure (in terms of the negative emotionality trait). They were disliked by people with the opposite characteristics in each trait. Not only did conscientious people dislike archetypal character hats, but they were also unwilling to wear them.

Participants scoring high in negative emotionality (i.e., emotionally unstable) also disliked, and in this case were also unwilling to wear, Classic Brimmed hats (Factor 5)—which conversely were liked by people emotionally stable, who were also willing to wear them. Emotionally unstable people were willing to wear Extravagant Highly Feminine Hats (that conversely emotionally stable people did not like to wear), even though an association between these types of hats and the negative emotionality trait was not found for liking.

Extroverts did not like both Extravagant Highly Feminine Hats and Archetypal Masculine Character Hats. This double result suggests that extroverts do not like hats that are too stereotypically marked.

Hats grouped in Factor 2, that is, Knitted Floppy Hats, showed no significant association with liking or willingness to wear, suggesting that preferences for these hats are relatively independent of broad personality traits. This is likely to be one of the reasons why they are so commonly used by young Italians, in addition to influences from fashion trends.

With regard to baseball caps (set 2), a greater number of associations were found between personality traits and the uncommon Extended Functional Protection Caps in Factor 1 in terms of both liking and willingness to wear, while only one association was found for the Casual Streetwear Style Caps in Factor 2. In other words, liking and willingness to wear common baseball cap models (such as the classic baseball cap, the trucker cap, the five-panel cap and the hip-hop cap) are minimally predicted by personality aspects. In this case, preference is likely to be more related to social factors, such as fashion trends, as well as the practical opportunity to use them in outdoor activities. Only two associations were found between personality traits and the Casual Streetwear Style Caps. One was with conscientiousness: the more conscientious a person is, the less they like these caps and the less likely they are to wear them. This latter result was found only for female participants. The other association was with open mindedness and willingness to wear: the more open-minded a person is, the less they like the idea of wearing these hats. This latter result was found only for male participants.

In contrast, preference for Extended Functional Protection Caps was extensively predicted by personality traits. In terms of liking, these hats were favored by open-minded participants and disliked by those scoring highly in agreeableness, conscientiousness and negative emotionality. In terms of willingness to wear them, a negative association was found between these hats and negative emotionality and extraversion in females. This suggests that introverted and emotionally stable women were more willing to wear them than extroverted and emotionally unstable women. A positive association with willingness to wear these hats was also found with open mindedness, but again only for females. While open-minded participants in general liked these hats, only open-minded women were willing to wear them.

The results of this study seem promising not only for advancing basic psychology research linking personality to aesthetic preference but also for stimulating new ideas in design studies and marketing research for customized product recommendation systems. The improvement of customized recommendation systems is certainly one of the main factors that has contributed to the expansion of e-commerce sales, which has become the main purchasing channel in the last 10 years (Chakraborty et al., 2021). A product recommendation system that is merely based on users’ previously purchased items and evaluations has the obvious limitation of not being able to provide accurate recommendations for new users, who lack profiling within the system (Paryudi et al., 2022). To overcome this and other limitations, the personality-aware recommender system (Dhelim et al., 2021) was developed. In this system, which is becoming widely common nowadays, customers’ personality type is assessed using either a personality questionnaire which customers have to answer during a registration phase, or an artificial intelligence personality recognition algorithm, based on data that are already available online and related to the social networks to which the customer belongs. The system then matches the customer’s personality type with relevant items by calculating matching probabilities between certain items and certain types of personalities. In this type of recommendation system, the degree of alignment between a product and a buyer is determined by linking textual descriptions of items with the personality types of the users who produce them or employing association models that focus on specific aspects of the products. The development of new research (like the study presented in this paper), which provides a direct assessment of product preferences with personality traits, may significantly expand the database on which the algorithms underlying these recommendation systems can ground their suggestions.

Despite the aforementioned potentialities, the study also has several limitations that future research should address. Firstly, the study does not answer whether there are specific visual, tactile, and expressive features of hats that are liked or disliked by particular personalities. Currently, we can only (approximately) predict which typologies of models would be liked or disliked (or worn or not worn) but we cannot identify the critical perceptual features that inform these choices.

Secondly, all of the participants in the study were Italian. Using a specific population is common in clothing preference research; for example, Hur et al. (2023) used a UK sample, Hur et al. (2025) used a UK and USA sample, Jegethesan et al. (2012) used Australian consumers, and Stolovy (2021) used Israeli women. While there is some evidence of the cross-cultural robustness of clothing preferences (e.g., Hur et al., 2025; Mair, 2018), it would be interesting to conduct the same study in other Western populations with fashion cultures different to that of Italy, using the same set of iconic models—as well as it would be interesting extending the study beyond Western culture.

Thirdly, our focus was on the shape of the hats, which is why we intentionally removed color information and only used grayscale images. However, we are aware that color can severely affect willingness to wear and purchase choices. Future studies might want to consider whether the associations that emerged in this study are robust in the presence of color information, and to what extent color might severely transform some of the patterns of preferences that emerged in the present study.

Fashion trends can be another intervening factor in participants’ choices, moderating the effect of personality traits. In our study, we have intentionally not focused on trendy models, but on a wide variety of models, with the aim of downsizing the role of this factor. However, we are aware that this does not guarantee the exclusion of fashion biases from participants’ responses—in fact, they may underlie the lack of association between personality traits and knitted floppy hats (Factor 2 in the iconic hat sample). This is another limitation of the study. We hope to see these limitations addressed in future studies.

## Figures and Tables

**Table 1:** Correlation between the ratings provided by each participant to the questions “How much do you like it?” and “How willing are you to wear it?” (ratings were given on a continuous scale of 0–100).

Hat Model	Pearson r	Hat Model	Pearson r
Balaclava	0.766 ***	News boy cap	0.787 ***
Baseball cap	0.837 ***	Panama hat	0.782 ***
Beret beanie	0.794 ***	Peak beanie	0.846 ***
Beret hat	0.774 ***	Pillbox 1	0.713 ***
Bobble beanie	0.848 ***	Pillbox 2	0.715 ***
Bolero hat	0.771 ***	Short beanie	0.855 ***
Cartwheel hat	0.716 ***	Tam hat	0.848 ***
Chullo	0.766 ***	Turban	0.767 ***
Cloche	0.677 ***	Audrey hat	0.686 ***
Cowboy hat	0.755 ***	Boater hat	0.732 ***
Deerstalker hat	0.700 ***	Bowler hat	0.679 ***
Fascinator hat	0.603 ***	Fedora hat	0.768 ***
Fisherman	0.850 ***	Homburg hat	0.728 ***
Flat cap	0.762 ***	Popeye hat	0.605 ***
Floppy hat	0.723 ***	Pork pie hat	0.744 ***
Long stoking cap	0.844 ***	Top hat	0.543 ***
Naked beanie	0.856 ***	Trilby hat	0.789 ***

Significance Codes: *p* < 0.001 (***).

**Table 2:** Standardized regression coefficients (β) referred to the SEM conducted to examine the extent to which the Big Five personality traits predicted the five hat-preference factors (for hats in set 1 and liking ratings).

Path		Estimate (β-Std.)	SE	z	*p*-Value
Flat/Urban Style Hats ←					
	Extraversion	−0.013	0.006	−0.236	0.814
	Agreeableness	−0.015	0.008	−0.286	0.775
	Conscientiousness	−0.086	0.006	−1.543	0.123
	Negative Emotionality	0.005	0.006	0.086	0.931
	Open Mindedness	0.237	0.007	4.618	<0.001 ***
Knitted Floopy Hats ←					
	Extraversion	−0.055	0.006	−0.999	0.318
	Agreeableness	0.058	0.007	1.213	0.225
	Conscientiousness	0.039	0.006	0.772	0.440
	Negative Emotionality	−0.030	0.005	−0.562	0.574
	Open Mindedness	0.053	0.007	0.961	0.337
Extravagant Highly Feminine Hats ←					
	Extraversion	−0.105	0.006	−2.054	0.040 *
	Agreeableness	−0.016	0.008	−0.303	0.762
	Conscientiousness	−0.030	0.006	−0.575	0.565
	Negative Emotionality	−0.087	0.005	−1.592	0.111
	Open Mindedness	0.161	0.006	3.258	0.001 **
Archetypal Masculine Character Hats ←					
	Extraversion	−0.170	0.007	−2.863	0.004 **
	Agreeableness	−0.131	0.008	−2.471	0.013 *
	Conscientiousness	−0.146	0.006	−2.759	0.006 **
	Negative Emotionality	−0.147	0.006	−2.532	0.011 *
	Open Mindedness	0.296	0.007	5.704	<0.001 ***
Classic Brimmed Hats ←					
	Extraversion	−0.056	0.006	−1.136	0.256
	Agreeableness	−0.050	0.007	−1.006	0.315
	Conscientiousness	−0.066	0.005	−1.353	0.176
	Negative Emotionality	−0.139	0.005	−2.800	0.005 **
	Open Mindedness	0.184	0.006	3.804	<0.001 ***

Significance Codes: *p* < 0.05 (*); *p* < 0.01 (**); *p* < 0.001 (***), The single-headed arrow from the personality traits to the specific hat factor represents a direct structural path, specifying that the personality traits are modeled as exogenous predictors exerting a directional effect on the outcome variable.

**Table 3:** Standardized regression coefficients (β) referred to the SEM conducted to examine the extent to which the Big Five personality traits predicted the five hat-preference factors (for hats in set 1 and willingness to wear ratings).

Path		Estimate (β-Std.)	SE	z	*p*-Value
Flat/Urban Style Hats ←					
	Extraversion	−0.032	0.007	−0.542	0.588
	Agreeableness	−0.056	0.008	−1.026	0.305
	Conscientiousness	−0.075	0.006	−1.380	0.168
	Negative Emotionality	−0.054	0.006	−0.878	0.380
	Open Mindedness	0.218	0.006	4.277	<0.001 ***
Knitted Floopy Hats ←					
	Extraversion	−0.034	0.006	−0.600	0.549
	Agreeableness	0.024	0.008	0.457	0.647
	Conscientiousness	0.049	0.006	0.921	0.357
	Negative Emotionality	0.018	0.005	0.337	0.736
	Open Mindedness	0.098	0.008	1.612	0.107
Extravagant Highly Feminine Hats ←					
	Extraversion	0.038	0.007	0.614	0.539
	Agreeableness	0.103	0.009	1.730	0.084
	Conscientiousness	0.064	0.006	1.137	0.255
	Negative Emotionality	0.117	0.005	2.210	0.027 *
	Open Mindedness	0.114	0.008	1.868	0.062
Archetypal Masculine Character Hats ←					
	Extraversion	−0.097	0.007	−1.647	0.100
	Agreeableness	−0.078	0.008	−1.427	0.154
	Conscientiousness	−0.154	0.006	−3.137	0.002 **
	Negative Emotionality	−0.098	0.006	−1.645	0.100
	Open Mindedness	0.214	0.007	3.749	<0.001 ***
Classic Brimmed Hats ←					
	Extraversion	−0.024	0.006	−0.492	0.623
	Agreeableness	−0.092	0.008	−1.761	0.078
	Conscientiousness	−0.040	0.005	−0.883	0.377
	Negative Emotionality	−0.120	0.005	−2.538	0.011 *
	Open Mindedness	0.182	0.006	3.708	<0.001 ***

Significance Codes: *p* < 0.05 (*); *p* < 0.01 (**); *p* < 0.001 (***), The single-headed arrow from the personality traits to the specific hat factor represents a direct structural path, specifying that the personality traits are modeled as exogenous predictors exerting a directional effect on the outcome variable.

**Table 4:** Correlation between liking and willingness to wear ratings referring to the Baseball Cap set (on a continuous scale of 0–100).

Hat Model	Pearson, r
Face saver	0.612 ***
Flap cap	0.818 ***
Trucker cap	0.855 ***
Army hat	0.828 ***
Iconic baseball cap	0.856 ***
Five panel cap	0.867 ***
Hip hop cap	0.825 ***
Visor cap	0.702 ***

Significance Codes: *p* < 0.001 (***).

**Table 5:** Standardized regression coefficients (β) referred to the SEM conducted to examine the extent to which the Big Five personality traits predicted the two hat-preference factors (for hats in set 2 and liking ratings).

Path		Estimate (β-Std.)	SE	z	*p*-Value
Extended Functional Protection Caps ←					
	Extraversion	−0.070	0.006	−1.438	0.150
	Agreeableness	−0.166	0.008	−3.240	0.001 **
	Conscientiousness	−0.126	0.006	−2.626	0.009 **
	Negative Emotionality	−0.228	0.005	−4.429	<0.001 ***
	Open Mindedness	0.204	0.006	4.656	<0.001 ***
Casual Streetwear Style Caps ←					
	Extraversion	−0.026	0.006	−0.493	0.622
	Agreeableness	0.050	0.007	1.026	0.305
	Conscientiousness	−0.119	0.006	−2.230	0.026 *
	Negative Emotionality	−0.080	0.005	−1.518	0.129
	Open Mindedness	−0.026	0.007	−0.500	0.617

Significance Codes: *p* < 0.05 (*); *p* < 0.01 (**); *p* < 0.001 (***), The single-headed arrow from the personality traits to the specific hat factor represents a direct structural path, specify-ing that the personality traits are modeled as exogenous predictors exerting a directional effect on the outcome variable.

**Table 6:** Standardized regression coefficients (β) referred to the SEM conducted to examine the extent to which the Big Five personality traits predicted the two hat-preference factors (for hats in set 2, and willingness to wear ratings).

		Males				Females			
Path		Estimate (β-Std.)	SE	z	*p*-Value	Estimate (β-Std.)	SE	z	*p*-Value
Extended Functional Protection Caps ←									
	Extraversion	0.047	0.012	0.429	0.668	−0.130	0.003	−2.143	0.032 *
	Agreeableness	−0.084	0.016	−0.750	0.453	−0.063	0.004	−1.078	0.281
	Conscientiousness	−0.025	0.011	−0.279	0.780	−0.103	0.003	−1.763	0.078
	Negative Emotionality	0.083	0.012	0.709	0.478	−0.287	0.004	−3.309	0.001 **
	Open Mindedness	0.138	0.015	1.245	0.213	0.133	0.004	1.973	0.049 *
Casual Streetwear Style Caps ←									
	Extraversion	0.093	0.012	0.885	0.376	−0.019	0.006	−0.327	0.744
	Agreeableness	0.030	0.015	0.298	0.766	0.072	0.008	1.240	0.215
	Conscientiousness	0.075	0.013	0.685	0.494	−0.127	0.006	−2.151	0.031 *
	Negative Emotionality	0.015	0.012	0.128	0.898	0.008	0.006	0.134	0.894
	Open Mindedness	−0.215	0.013	−2.175	0.030 *	0.064	0.007	1.066	0.286

Significance Codes: *p* < 0.05 (*); *p* < 0.01 (**), The single-headed arrow from the personality traits to the specific hat factor represents a direct structural path, specify-ing that the personality traits are modeled as exogenous predictors exerting a directional effect on the outcome variable.

## Data Availability

Data are available at the following link: https://osf.io/kazf8/?view_only=152c6094ddc74d2b8ad148dd0ed89e8c (accessed on 25 October 2024).
